# Oral Ondansetron versus Domperidone for Acute Gastroenteritis in Pediatric Emergency Departments: Multicenter Double Blind Randomized Controlled Trial

**DOI:** 10.1371/journal.pone.0165441

**Published:** 2016-11-23

**Authors:** Federico Marchetti, Maurizio Bonati, Alessandra Maestro, Davide Zanon, Francesca Rovere, Alberto Arrighini, Egidio Barbi, Paolo Bertolani, Paolo Biban, Liviana Da Dalt, Andrea Guala, Elisa Mazzoni, Anna Pazzaglia, Paolo Francesco Perri, Antonino Reale, Salvatore Renna, Antonio Francesco Urbino, Enrico Valletta, Antonio Vitale, Tiziana Zangardi, Antonio Clavenna, Luca Ronfani, on behalf of the SONDO (Study ONdansetron vs DOmperidone) Investigators

**Affiliations:** 1 Department of Pediatrics, Institute for Maternal and Child Health - IRCCS "Burlo Garofolo”, Trieste, Italy; 2 Laboratory for Mother and Child Health, Department of Public Health, IRCCS - Istituto di Ricerche Farmacologiche "Mario Negri", Milano, Italy; 3 Pharmacy and Clinical Pharmacology, Institute for Maternal and Child Health - IRCCS "Burlo Garofolo”, Trieste, Italy; 4 Pediatric Emergency Department, Presidio Ospedale dei Bambini, A.O. Spedali Civili, Brescia, Italy; 5 Pediatric Emergency Department, Institute for Maternal and Child Health - IRCCS "Burlo Garofolo”, Trieste, Italy; 6 Pediatric Unit, Department of Medical and Surgical Sciences for Mothers, Children and Adults, University of Modena and Reggio Emilia, Modena, Italy; 7 Pediatric Emergency Department, Azienda Ospedaliera Universitaria Integrata, Verona, Italy; 8 Department of Pediatrics, Treviso Hospital, Treviso, Italy; 9 Department of Pediatrics, Ospedale Castelli, Verbania, Italy; 10 Department of Pediatrics, Ospedale Maggiore, Bologna, Italy; 11 Emergency Department, Pediatric Hospital A. Meyer, Firenze, Italy; 12 Department of Pediatrics, Ospedale di Macerata, Macerata, Italy; 13 Emergency Department, Ospedale Pediatrico Bambino Gesù, IRCCS, Roma, Italy; 14 Emergency Room and Emergency Medicine Division, G. Gaslini Institute, Genova, Italy; 15 Emergency Department, Ospedale Infantile Regina Margherita, Torino, Italy; 16 Department of Pediatrics, Ospedale G.B. Morgagni - L. Pierantoni, Forlì, Italy; 17 Department of Pediatrics and Pediatric Emergency, "San Giuseppe Moscati" National Hospital (AORN), Avellino, Italy; 18 Pediatric Emergency Department, Azienda Ospedaliera - University of Padova, Padova, Italy; 19 Clinical Epidemiology and Public Health Research Unit, Institute for Maternal and Child Health - IRCCS "Burlo Garofolo”, Trieste, Italy; National Cancer Institute, UNITED STATES

## Abstract

The use of antiemetics for vomiting in acute gastroenteritis in children is still a matter of debate. We conducted a double-blind randomized trial to evaluate whether a single oral dose of ondansetron vs domperidone or placebo improves outcomes in children with gastroenteritis. After failure of initial oral rehydration administration, children aged 1–6 years admitted for gastroenteritis to the pediatric emergency departments of 15 hospitals in Italy were randomized to receive one oral dose of ondansetron (0.15 mg/kg) or domperidone (0.5 mg/kg) or placebo. The primary outcome was the percentage of children receiving nasogastric or intravenous rehydration. A p value of 0.014 was used to indicate statistical significance (and 98.6% CI were calculated) as a result of having carried out two interim analyses. 1,313 children were eligible for the first attempt with oral rehydration solution, which was successful for 832 (63.4%); 356 underwent randomization (the parents of 125 children did not give consent): 118 to placebo, 119 to domperidone, and 119 to ondansetron. Fourteen (11.8%) needed intravenous rehydration in the ondansetron group vs 30 (25.2%) and 34 (28.8%) in the domperidone and placebo groups, respectively. Ondansetron reduced the risk of intravenous rehydration by over 50%, both vs placebo (RR 0.41, 98.6% CI 0.20–0.83) and domperidone (RR 0.47, 98.6% CI 0.23–0.97). No differences for adverse events were seen among groups. In a context of emergency care, 6 out of 10 children aged 1–6 years with vomiting due to gastroenteritis and without severe dehydration can be managed effectively with administration of oral rehydration solution alone. In children who fail oral rehydration, a single oral dose of ondansetron reduces the need for intravenous rehydration and the percentage of children who continue to vomit, thereby facilitating the success of oral rehydration. Domperidone was not effective for the symptomatic treatment of vomiting during acute gastroenteritis.

## Introduction

Acute gastroenteritis (AGE) is the main cause of acute vomiting in children under the age of 3 years and one of the most important reasons for admission to the pediatric emergency department (ED) and the hospital [1,2]. In the USA, 1.5 million children under 5 years are diagnosed with AGE annually and this condition accounts for 13% of all hospital admissions [1]. The most frequent complication is dehydration. In Europe, at least 230 deaths and over 87,000 hospitalizations of children under 5 years of age are reported every year.[3]

In the initial phase of AGE, vomiting is reported in 75% of children with rotavirus infection [4], and is distressing for both patients and their families. Vomiting is a direct cause of fluid loss and can also hamper successful treatment with oral rehydration solution (ORS). Symptomatic pharmacological treatment for vomiting is still a matter of debate and is not systematically included in current practice recommendations for pediatric AGE [5–7]. Physicians and parents in ED favor intravenous fluid therapy (IVT) for mild or moderate dehydration when vomiting is the major symptom [8, 9]. Thus, effective antiemetic treatment would lead to an important reduction in the use of IVT.

Various antiemetic agents are available and are often used off-label to prevent or reduce vomiting in children with AGE [10, 11]. In France, Spain, Italy and in other European countries, the dopamine receptor antagonist domperidone is the preferred antiemetic treatment [12]. Ondansetron is administered to only a small proportion of children and its use varies significantly among institutions [13, 14].

Literature evaluating the efficacy of symptomatic drugs in reducing acute vomiting for pediatric AGE focuses mainly on ondansetron [15–19]. Evidence exists that ondansetron compared with placebo increases the proportion of patients with cessation of vomiting, reduces the immediate hospital admission rate and the need for IVT. However, not all of these studies evaluate first-line oral rehydration therapy (ORT) during hospital stay before the administration of the antiemetic [18], and an adequate comparative evaluation between domperidone and ondansetron is missing [4, 20]. Concerning the use of domperidone, only few studies are available with small sample sizes, low methodological quality, and inconsistent results [4, 15, 17, 18–23].

The aim of the current trial was to assess whether the oral administration of ondansetron vs domperidone or placebo, after a first attempt with ORS, prevents IVT or nasogastric rehydration in children with vomiting during AGE.

## Methods

### Study design

This prospective, multicenter, double-blind randomized controlled trial involved children admitted to 15 pediatric EDs in Italy.

The study was coordinated by the Institute for Maternal and Child Health-IRCCS Burlo Garofolo (Trieste) and by the Maternal and Child Health Laboratory-IRCCS-Istituto di Ricerche Farmacologiche Mario Negri (Milan). The protocol (S1 Protocol), which has been previously published [24], obtained the ethical approval by the Independent Bioethic Committee of the coordinating center (IRCCS Burlo Garofolo, Trieste Italy) on November 8, 2010 (Approval Number: S-115) and, subsequently, by the ethic committees of each participating center (Comitato Etico dell’Azienda Ospedaliera Spedali Civili di Brescia; Comitato Etico della Provincia di Modena; Comitato Etico per la sperimentazione dell’Azienda Ospedaliera Istituti Ospitalieri di Verona; Comitato Etico per la sperimentazione clinica della Provincia di Treviso; Comitato Etico dell’Azienda Ospedaliera Universitaria Maggiore della Carità di Novara; Comitato Etico della AUSL di Bologna; Comitato Etico locale per la sperimentazione dei farmaci dell’A.O. Universitaria Anna Meyer, Firenze; Comitato Etico interzonale della ASUR Zona Territoriale 8 di Civitanova Marche e Zona Territoriale 9 di Macerata; Comitato Etico per la sperimentazione clinica dell’IRCCS Ospedale pediatrico Bambino Gesù di Roma; Comitato Etico dell’IRCCS Istituto Giannina Gaslini, Genova; Comitato Etico dell’Azienda Ospedaliera OIRM S. Anna di Torino; Comitato Etico di Area Vasta Romagna di Cesena e Istituto scientifico romagnolo per lo studio e la cura dei tumori di Meldola, Forlì; Comitato Etico dell’Azienda Ospedaliera S. Giuseppe Moscati di Avellino; Comitato Etico per la sperimentazione dell’Azienda Ospedaliera di Padova). The trial was registered before participant recruitment began at Clinicaltrials.gov (NCT01257672) and at the European Union Clinical Trials Register EudraCT (Number: 2010-019787-36).

The Italian Medicines Agency (AIFA) funded the trial, including the reimbursement of the costs of production of the drugs by Monteresearch S.r.l (www.monteresearch.it), a pharmaceutical development service licensed by AIFA to produce and manage medical products for clinical trials according to Good Manufacture Practice (GMP), with no role in trial design and conduction. The trial received no commercial funding. A written informative document was handed to parents or legal surrogate prior to enrollment and contained detailed information on the study, the burden of the intervention, including the length of ED stay in case of participation and the possible adverse events. Written informed consent was obtained from each child’s parent or legal surrogate.

A multidisciplinary steering committee (1 epidemiologist, 1 clinician, 1 pharmaco-epidemiologist) was established to monitor the data, ensure patient safety and act as reference for Participants Units. The members of the committee were not directly involved in the actual field work.

### Patients

Eligible children were aged 1 to 6 years, presenting with vomiting, with or without diarrhea, with a presumptive clinical diagnosis of AGE and more than three non-bilious and non-bloody vomiting episodes (S1 Text) within the previous 24 hours.

The exclusion criteria were the use of antiemetics or antidiarrheal drugs in the 6 hours prior to access to ED, underlying chronic diseases (i.e., malignancy, gastroesophageal reflux, migraine, renal failure, hypoalbuminemia, liver disease), severe dehydration defined by a standard clinical score of ≥18 for children 12–24 months or ≥16 for children ≥24 months of age (S1 Table) [25], known hypersensitivity to ondansetron or domperidone, previous enrollment in the study, concomitant use of drugs that prolong the QT interval, language barriers or inability to perform the telephone follow-up. The last two exclusion criteria were added after discussion with the Bioethic Committee of the Coordinating Center (Trieste).

### Randomization and masking

Patients were randomly assigned in fixed blocks of nine to receive ondansetron or domperidone or placebo in a 1:1:1 ratio. The randomization list was generated using the STATA software and was stratified according to participating centers. The randomization procedure was centralized. The randomization sequence was transmitted to the pharmaceutical development service (Monteresearch S.r.l.), that prepared and sent directly to participating hospitals, active drugs and placebo in closed, opaque and consecutively numbered bags. Drug preparations were indistinguishable by taste, odor and appearance. A syrup was preferred to other possible formulations (i.e. tablets) because it allows for the preparation of solutions at different concentrations which makes it possible to administer the same volume, based only on the child's weight (ml/kg), regardless of the allocation group. Each bag contained a graduated drug dispenser. For each randomization the amount of syrup allowed for a second administration in vomiting children within 15 min of the first dose. After confirmation of first-line ORT failure, the next available bag containing the drug preparation was opened and a weigh-appropriate dose was administered to the patient. Study investigators and participants were unaware of the randomization list and blind to the pharmaceutical preparations assigned.

### Procedures

After checking for inclusion and exclusion criteria, a first ORT attempt was carried out following the standard protocol (S2 Text). In case of failure of the initial ORS administration, defined as vomiting after ORS or fluid refusal after three attempts, patients were randomized to receive an oral administration of:

1. ondansetron syrup (0.15 mg/Kg of body weight);
2. domperidone syrup (0.5 mg/Kg of body weight);
3. placebo syrup.

The dosages of ondansetron and domperidone were those indicated by the Summary of Product Characteristics. Children who vomited again within 15 minutes of receiving the drug, were given a second dose. A new ORT attempt was carried out 45 to 60 minutes after the first treatment. Patients were reassessed at 30 minute intervals for a minimum of 6 hours and data was collected at each assessment. Forty-eight hours after discharge, a blinded research assistant phoned the child’s family to assess, using a standard form, the evolution of AGE, the need for hospitalization or readmission to the ED and the final outcome.

### Outcomes

According to the published protocol [24], the primary outcome was the percentage of patients who were administered nasogastric or intravenous rehydration after symptomatic oral treatment failure, defined as vomiting or fluid refusal after the second ORT attempt. Secondary outcomes were: a) the percentage of subjects remaining in ED for observation stay for more than 6 hours; b) the percentage of subjects requiring hospital admission during the ED stay; c) subjects with episodes of vomiting and number of episodes in the 3 treatment groups during the ED stay and during the 48-hour follow-up period; d) the percentage of subjects presenting adverse events during ED stay and during the 48-hour follow-up period. Two further outcomes were evaluated: the rate of success at the second ORT attempt and the percentage of subjects requiring laboratory tests during ED stay. Moreover, the subjects with episodes of diarrhea and the number of episodes in the 3 treatment groups were evaluated both during ED stay and during the 48-hour follow-up period.

The information on the number of vomiting and diarrhea episodes after ED discharge was referred to the last 24 hours of follow-up with the aim of verifying real symptom resolution.

### Safety profile

In case of serious or medically relevant clinical adverse events or abnormal laboratory test values registered during the course of the study or in the post-treatment period, the investigators were obliged to inform the Coordinating Units immediately. The Coordinating Units were responsible for sending the reports on suspected unexpected serious adverse reactions to all participating investigators, to AIFA and to the Ethics Committees, in accordance with international and Italian laws and regulations as well as with International Conference on Harmonisation of Technical Requirements for Registration of Pharmaceuticals for Human Use (ICH)/Good Clinical Practice (GCP) guidelines.

### Statistical analysis

To estimate sample size we initially referred to the Roslund RCT that implemented a similar protocol, enrolling subjects with AGE who failed initial ORS administration in the ED [26]. We estimated, using the Fleiss method with continuity correction, that the enrollment of 540 children (i.e. 180 patients in each arm) would provide the study with a statistical power of 80% to detect a change from 50% in placebo group to 35% in domperidone group and 20% in ondansetron group in the proportion of children receiving nasogastric or IVT, given a two-sided type I error of 0.05. Given the lack of efficacy estimates, the effect of domperidone was estimated as intermediate between ondansetron and placebo.

In the original protocol no interim analyses were planned. However, given the difficulty to enroll patients due to the unexpected success of the first ORT, in accordance with the study Sponsor (AIFA), in June 2013 we amended the protocol adding 2 blind interim analyses and 1 possible final analysis following the O'Brien-Fleming criteria [27]. The first interim analysis was planned for July 2013, two years after the enrollment of the first subject in the study (critical value p = 0.0005). A second interim analysis was planned for November 4^th^ 2013 (the expected date for the end of the enrollment) if: 1) the first analysis had not achieved the necessary significance, or 2) the initially estimated sample size had not been reached. The second interim analysis (critical value p = 0.014) allowed us to close the study with a final sample size of 356 children (Table A and Table B in S2 Table). The decision was taken after consultation with the study steering committee. The two interim analysis were carried out at the Epidemiology and Biostatistics Unit of the coordinating center by a statistician not involved in the study and blinded to the allocation group.

Numbers, percentages and, when appropriate, relative risks and confidence intervals (CI) are presented for categorical data and medians and interquartile ranges (IQR) for continuous data. For categorical outcomes, differences between ondansetron vs placebo and ondansetron vs domperidone were evaluated using the chi-square test and for continuous outcomes using the non-parametric Mann-Whitney U test, since data were not normally distributed. All p values and estimates of treatment effects were based on separate comparisons, so no adjustments were made for multiple comparisons. Analyses were performed with SPSS software (version 21.0) according to the intention-to-treat principle. All p values are two-sided. According to the O'Brien-Fleming criteria, a p value of less than 0.014 was used to indicate statistical significance and 98.6% CI were calculated.

## Results

**Fig 1:**
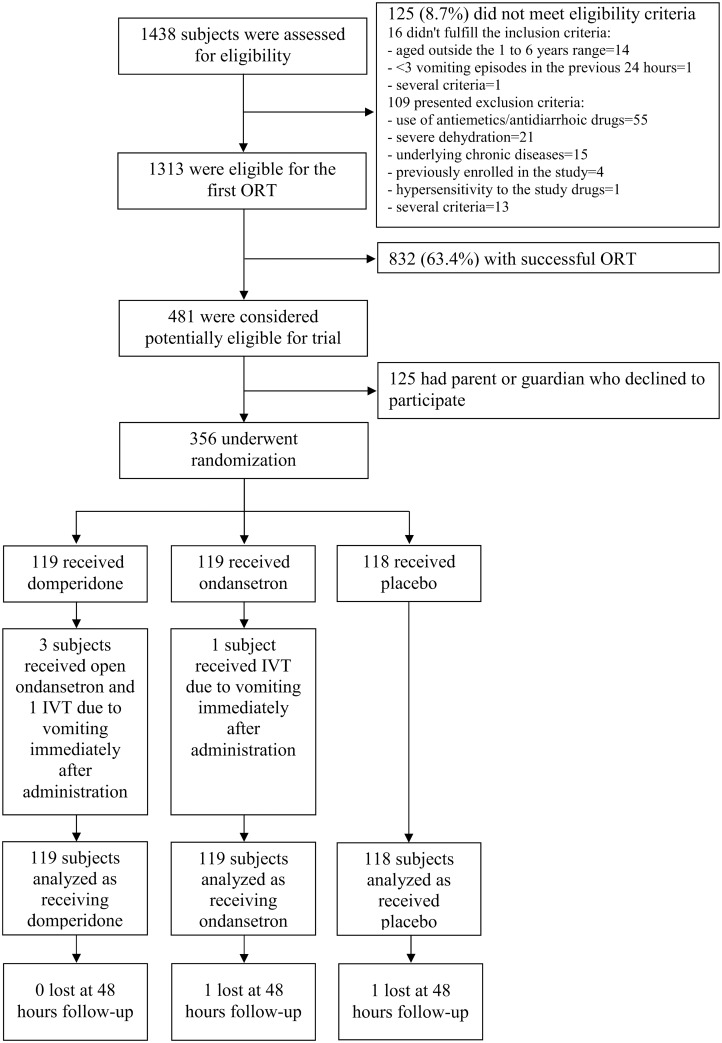
Study flow chart: assessment, randomization, and follow-up.

Participants were recruited between July 7, 2011 (first randomization) and November 3, 2013 (last follow-up). A total of 1438 children with AGE who had accessed the 15 EDs were assessed, 1313 were eligible for the first ORT attempt which was successful for 832 (63.4%). Of the remaining 481 children, 125 (25.9%) were excluded because parents or guardians did not give their consent while 356 were randomly assigned to the study groups: 119 to domperidone, 119 to ondansetron and 118 to placebo (Fig 1). The baseline characteristics of the groups were similar (Table 1).

**Table 1:** Baseline Characteristics of the Study Patients.

	Ondansetron (n = 119)	Domperidone (n = 119)	Placebo (n = 118)
Age (years)	3.1 (2.1–4.2)	3.2 (1.9–4.6)	3.3 (2.1–4.7)
Male	57 (47.9%)	65 (54.6%)	54 (45.8%)
Weight (Kg)	14.2 (11.5–18.2)	14.5 (11.7–17.5)	15.5 (12.7–18.3)
Height (cm)§	98.5 (89.0–110.0)	99.0 (86.8–110.0)	99.0 (88.5–110.5)
Duration of vomiting before enrollment (hours)	10.0 (6.0–24.0)	9.0 (5.0–18.0)	12.0 (7.0–21.0)
Number of episodes of vomiting			
in the last 24 hours	7.0 (5.0–10.0)	8.0 (6.0–10.0)	8.0 (5.8–10.0)
in the last 6 hours	5.0 (3.0–7.0)	6.0 (4.0–7.0)	5.0 (3.0–7.3)
Presence of diarrhea	51 (42.9%)	47 (39.5%)	49 (41.5%)
Drugs taken in the last 6 hours	19 (16.0%)	21 (17.6%)	17 (14.4%)
Total dehydration score	8.0 (7.0–9.0)	8.0 (7.0–9.0)	8.0 (7.0–9.0)
Dehydration score by category			
no dehydration	63 (52.9%)	69 (58.0%)	59 (50.0%)
mild to moderate dehydration*	56 (47.1%)	50 (42.0%)	59 (50.0%)
Subjects needing a second dose because of vomiting within 15 minutes	8 (6.7%)	22 (18.5%)	11 (9.3%)

Data are n (%) or median (IQR).

^§^Available for 277 subjects (94 domperidone, 90 ondansetron, 93 placebo).

*Score 10–17 if age <24 months; score 8–15 if age ≥24 months

All the randomized subjects received the first dose of the study medication. For the majority of patients (315/356, 88.5%) vomiting did not interfere with the first administration, whereas 22 patients receiving domperidone (18.5%), 8 ondansetron (6.7%) and 11 placebo syrup (9.3%) needed a second dose within 15 minutes after the first one. Five children (four in the domperidone and one in the ondansetron group) vomited immediately after the second dose. Three of these received one dose of open-label ondansetron, and two were treated with intravenous rehydration. According to the intention to treat approach, these children were considered to belong to their respective randomization group (Fig 1). The average amount of syrup was 3.8 ml (SD 1.2) for ondansetron, 3.8 ml (SD 1.1) for domperidone, and 3.9 ml (SD 1.0) for placebo (p = 0.66).

No patient received nasogastric rehydration. Intravenous treatment was administered to 78 out of 356 children (21.9%): 14 in the ondansetron group (11.8%), 30 (25.2%) in the domperidone group, and 34 (28.8%) in placebo group. Ondansetron led to a relative risk reduction of IVT by over 50%, both vs domperidone (RR 0.47, 98.6% CI 0.23 to 0.97) and placebo (RR 0.41, 98.6% CI 0.20 to 0.83) (Table 2). The number needed to treat (NNT) for ondansetron vs domperidone was 8 (95% CI from 5 to 28) and 6 (95% CI from 4 to 15) vs placebo.

**Table 2:** Outcome measures during Emergency Department stay.

	Ondansetron (n = 119)	Domperidone (n = 119)	Placebo (n = 118)	Ondansetron vs Placebo	Ondansetron vs Domperidone
				RR (98.6% CI), P-value	RR (98.6% CI), P-value
Subjects receiving nasogastric or intravenous rehydration (primary outcome)	14 (11.8%)	30 (25.2%)	34 (28.8%)	0.41 (0.20 to 0.83), p = 0.001	0.47 (0.23 to 0.97), p = 0.008
Subjects needing observation stay for more than 6 hours for the same illness	20 (16.8%)	37 (31.1%)	39 (33.1%)	0.51 (0.28 to 0.92), p = 0.004	0.54 (0.30 to 0.99), p = 0.01
Subjects requiring hospital admission	10 (8.4%)	16 (13.4%)	20 (16.9%)	0.50 (0.20 to 1.22), p = 0.05	0.63 (0.24 to 1.60), p = 0.21
Subjects with episodes of vomiting during ED stay	20 (16.8%)	53 (44.5%)	49 (41.5%)	0.41 (0.23 to 0.71), p<0.0001	0.38 (0.22 to 0.66), p<0.0001
Number of episodes of vomiting during ED stay	1.0 (1.0–2.0)	2.0 (1.0–3.0)	2.0 (1.0–3.0)	p = 0.02	p = 0.04
Success at second ORT attempt	107 (89.9%)	78 (65.5%)	76 (64.4%)	1.40 (1.16 to 1.68), p<0.0001	1.37 (1.15 to 1.64), p<0.0001
Subjects requiring laboratory tests*	17 (14.3%)	31 (26.1%)	37 (32.2%)	0.44 (0.23 to 0.85), p = 0.001	0.55 (0.28 to 1.07), p = 0.02
Subjects with episodes of diarrhea during ED stay	33 (27.7%)	26 (21.8%)	20 (16.9%)	1.64 (0.88 to 3.04), p = 0.05	1.27 (0.72 to 2.22), p = 0.29
Number of episodes of diarrhea during ED stay	2.0 (1.5–4.5)	1.0 (1.0–2.0)	1.5 (1.0–2.0)	p = 0.02	p = 0.004

Data are n (%), median (IQR) or Relative Risk (98.6% Confidence Interval). ED = Emergency Department.

*Available for 353 subjects

A stratified analysis showed no effect of the dehydration status (no vs mild to moderate dehydration) on the primary study outcome.

Ondansetron halved the risk of the observation stay exceeding 6 hours versus both domperidone and placebo while the number of patients admitted to hospital from the ED was similar in all three groups (Table 2). Ondansetron significantly reduced the number of subjects with episode of vomiting during ED stay and led to greater success of the second attempt with ORS vs domperidone and placebo; furthermore, ondansetron significantly reduced the need for laboratory tests compared with placebo (Table 2). No statistically significant difference was seen for the occurrence of diarrhea while the median number of diarrhea episodes was higher in the ondansetron group (2.0 episodes vs 1.0 in domperidone and 1.5 in placebo group).

After discharge, no statistically significant differences were seen among the three groups for subjects readmitted in ED for the same illness, subjects with episodes of vomiting and diarrhea in the 48 hours follow-up and number of episode of vomiting and diarrhea in the last 24 hours of follow-up (Table 3).

**Table 3:** Outcome measures at telephone follow-up.

	Ondansetron (n = 118)	Domperidone (n = 119)	Placebo (n = 117)	Ondansetron vs Placebo	Ondansetron vs Domperidone
				RR (98.6% CI), P-value	RR (98.6% CI), P-value
Subjects readmitted to ED for the same illness after discharge	11 (9.3%)	12 (10.1%)	13 (11.1%)	0.84 (0.32 to 2.18), p = 0.65	0.92 (0.35 to 2.45), p = 0.84
Subjects with episodes of vomiting in the follow-up period of 48 hours	36 (30.5%)	27 (22.7%)	41 (35.0%)	0.87 (0.55 to 1.38), p = 0.46	1.35 (0.79 to 2.30), p = 0.17
Number of episodes of vomiting in the last 24 hours of follow-up	1.0 (1.0–3.3)	2.0 (1.0–3.0)	1.5 (1.0–3.1)	p = 0.87	p = 0.46
Subjects with episodes of diarrhea in the follow-up period of 48 hours	49 (41.5%)	51 (42.9%)	44 (37.6%)	1.10 (0.74 to 1.64), p = 0.54	0.97 (0.67 to 1.41), p = 0.84
Number of episodes of diarrhea in the last 24 hours of follow-up	3.0 (2.0–4.0)	3.0 (2.0–4.0)	3.0 (1.0–5.0)	p = 0.31	p = 0.32

Data are n (%), median (IQR) or Relative Risk (98.6% Confidence Interval). ED = Emergency Department

No serious adverse event was observed. A total of 13 patients had one mild adverse effect: 6 after ondansetron, 5 after domperidone, and 2 after placebo. Episodes of drowsiness, asthenia, irritability, diarrhea or abdominal pain were common to all the three investigated groups (S3 Table). In one case (ondansetron group) the blinding was opened following an adverse event.

## Discussion

Our study indicates a 63.4% success rate of the first attempt with ORS in over 1300 children with AGE without severe dehydration. This means that, in an ED setting, 6 out of 10 children aged 1–6 years with vomiting due to AGE and no or mild to moderate dehydration, can be successfully treated with oral rehydration solution alone, without the need for drugs. This finding is consistent with the estimates of the Cochrane review [17, 18].

In children who continue to vomit after the first ORT attempt, a single oral dose of ondansetron improves the chances of success of ORT. Ondansetron reduces by over 50% the number of patients requiring IVT vs both domperidone and placebo (Table 2), in agreement with the results of other RCTs [15–19, 25]. Our results provide clear evidence of benefit of ondansetron also with respect to the other study outcomes. Hospital admission rates are lower in the ondansetron group vs both domperidone and placebo. Differences among groups did not reach the statistical significance of the meta-analysis (RR 0.41; 95% IC 0.29 to 0.59) [18]. In the present study, the need for observation stay to last more than six hours is statistically significantly lower in the ondansetron group compared with the domperidone and placebo groups.

In agreement with the results of other RCTs, no difference in the percentage of patients readmitted to the ED within 48 hours of discharge was seen among the three groups. This percentage is lower than the one reported in Freedman's RCT and similar to that reported in another study [25, 28].

Only a few mild adverse events occurred after ondansetron administration, all of them resolved quickly without any consequence for the children. In particular, although the ondansetron group presented a higher median number of episodes of diarrhea (one additional episode on average), this increase is smaller than the one described in other RCTs [17, 18], and seems to have no clinical relevance, especially when weighed against the drug’s significant effect on reduction of vomiting. Unfortunately, our study does not have the statistical power to detect rare but serious adverse events, such as cardiac arrhythmias, and further studies, i.e. post-marketing surveillances, should be carried out to address this issue. Although outside the context of diarrhea, the FDA black box alert published in September 2011, recommends electrocardiogram monitoring in patients with potential “electrolyte abnormalities” receiving ondansetron, due to the risk of developing prolongation of the QT interval which can lead to an abnormal and potentially fatal heart rhythm, including Torsade de Pointes [4]. However, there is evidence that routine ECG and electrolyte screening before the administration of a single dose of oral ondansetron in children without known risk factors (i.e. history of arrhythmias, concomitant use of QT-prolonging drugs) are not necessary [29].

Domperidone did not appear to be superior in any of the primary and secondary outcomes (Table 2). The available evidence of the efficacy of domperidone consist of few studies with small sample sizes, low methodological quality and inconsistent results [4, 15, 17–23]. The only randomized trial comparing oral domperidone with placebo showed that the drug, used in combination with ORT, does not reduce vomiting in the early stages of AGE [23].

We chose to include domperidone in our RCT since it is commonly prescribed to children with gastroenteritis in several countries, including Italy [10–12, 23, 30], and is licensed in Europe for the “treatment of nausea and vomiting”, also in the pediatric population, despite the lack of evidence on efficacy. Recently the authorization for the use of domperidone for these clinical conditions has been subjected to restrictions because of the possible risks of severe arrhythmias, particularly in the case of drug overdose [4, 31]. Furthermore, case reports and post marketing surveillances have reported the occurrence of extrapyramidal reactions associated with the use of domperidone [32, 33].

The main limitation of our study is the premature closure of the enrollment, and the consequent failure to reach the initially estimated sample size. This has already been described for publicly-funded trials in the UK [34]. In our study this was due to the success of the first ORT which made it difficult to have patients to randomize and to the non-occurrence of the annual rotavirus diarrhea outbreak during the second year of the study. Furthermore, in the absence of literature evidence, the sample calculation of the efficacy of domperidone was estimated to be intermediate between ondansetron and placebo, but the study findings did not confirm this hypothesis. However, our RCT is the largest carried out on this topic so far.

The use of placebo in our RCT could be questioned. When the study started, available evidence, albeit weak and unreliable [17], suggested the efficacy of ondansetron for symptomatic treatment of vomiting during AGE in children but a proper evaluation of domperidone, largely used in clinical practice, was lacking. Furthermore, at the time, no formal indications for the use of ondansetron in the treatment of AGE was given by clinical practice guidelines. Consequently, and in accordance with the document on ethical considerations for clinical trials in the pediatric population [35], we felt that the use of placebo was legitimate. This matter was also discussed by the Bioethic Committee of the coordinating centre before the approval of the study protocol.

Our trial included a large number of patients with a limited age range (1–6 years), with no or mild to moderate dehydration. This reflects the actual clinical setting in Italy. The selected age range allowed us to enroll most of the children with community acquired AGE, which is prevalent in younger children, and to ensure an adequate safety profile, since ondansetron is approved for vomiting (after chemotherapy) in children over 6 months. This study contributes to provide evidence for better management of this young population characterized by high incidence of AGE associated to dehydration, and scanty evidence of effective therapeutic approaches.

Our study presents several strengths. First, the complete independence of the study, which was made possible by the public funding received from AIFA. Second, the initial use of ORS before administering the drugs and the inclusion in the trial only of children who had failed this first attempt. This made it possible to adequately evaluate the role of the drugs under study and to confirm in a field study the role and applicability of ORT in children with AGE without severe dehydration.

The results of our study have relevant implications for practice. Our trial fully confirms the results of the most recent systematic review on the use of ondansetron which suggests that the clinical practice guidelines for the treatment of children with AGE should be revised to include the use of a single dose of oral ondansetron in the case of continued vomiting after a first attempt with ORS [18]. This simple intervention reduces the need for IVT and laboratory tests, the discomfort of vomiting and the time spent in the ED. Currently, some guidelines, in particular those from North America [7], already suggest the use of ondansetron in pediatric emergency departments in infants and children between six months and 12 years of age, while others, including ESPGHAN and NICE [4, 6], are more conservative as a result of the FDA warning on the potentially severe adverse effects of ondansetron.

In view of the ineffectiveness demonstrated in the present trial, the inconclusive results of previous studies and the possible side effects reported in literature, domperidone should not be used for the symptomatic treatment of vomiting as a consequence of AGE in children.

The aims of our work did not include a pharmacoeconomic analysis, but a previous study showed that the administration of oral ondansetron to children with dehydration and vomiting secondary to AGE led to significant monetary savings compared to a no-ondansetron policy [36]. Appropriate strategies are needed to successfully incorporate oral ondansetron into clinical practice in order to maximize its potential benefits [37]. Further studies are needed to understand if, in the real context of ED care, the use of ondansetron in children at high risk of dehydration can effectively reduce the number of cases receiving IVT. Indeed, despite the increasing use of ondansetron over the years in the United States and Canada, the percentage of children requiring IVT doesn’t seem to have decreased [38].

In conclusion, our trial showed that, in a context of emergency care, 6 out of 10 children aged 1–6 years with vomiting due to AGE and without severe dehydration can be managed effectively with the administration of ORS alone. In children who continue to vomit or refuse ORT, a single oral dose of ondansetron reduces the need for IVT, the percentage of children who vomit and the number of episodes of vomiting, thereby facilitating the success of ORT. A small, not clinically relevant, increase in the number of episodes of diarrhea was observed in the ondansetron group. Domperidone was not effective for the symptomatic treatment of vomiting during AGE.

## Supporting Information

S1 CONSORT Checklist CONSORT 2010 checklist of information to include when reporting a randomised trial. (DOC)

S1 Dataset (XLS)

S1 Protocol Original and final study protocol. (DOC)

S1 Table Dehydration clinical score. (DOC)

S2 Table Interim analyses results for the main study outcome (subjects needing nasogastric or intravenous rehydration). (DOC)

S3 Table Adverse events. (DOC)

S1 Text Definitions of acute gastroenteritis and vomiting. (DOC)

S2 Text Study standard protocol of oral rehydration solution (ORS) administration. (DOC)
