# Supplementary material for: Oral Ondansetron versus Domperidone for Acute Gastroenteritis in Pediatric Emergency Departments: Multicenter Double Blind Randomized Controlled Trial
Source: PLoS One. 2016 Nov 23;11(11):e0165441. doi: 10.1371/journal.pone.0165441 (PMC5120790; doi:10.1371/journal.pone.0165441)
Supplement: S1 Protocol — (DOC) [file pone.0165441.s003.doc]

**S1 Protocol**

**Original and final study protocol**

This supporting information file contains the following items:

1. Original protocol

2. Final protocol

3. Summary of changes

**1.** **Original study protocol[[1]](#footnote-2)**

**ORAL ONDANSETRON VS DOMPERIDONE FOR SYMPTOMATIC TREATMENT OF VOMITING DURING ACUTE GASTROENTERITIS IN CHILDREN: MULTICENTRE RANDOMIZED CONTROLLED TRIAL**

**BACKGROUND**

**Acute Gastroenteritis**

Acute gastroenteritis (AG) is the main cause of acute vomiting in children aged under 3 years and one of the most important reasons for access to the emergency department (ED) and admission to hospital.

In USA 1.5 millions of children under 5 years are diagnosed AG annually and 13% of these children are admitted to the hospital (1). In Italy “esophagitis, gastroenteritis and a miscellaneous of digestive apparatus diseases” (DRG 184) results to be the most important cause of hospital admission in paediatric age (2). The World Health Organization (WHO), the American Academy of Pediatrics (AAP), and the European Society for Paediatric Gastroenterology, Hepatology, and Nutrition (ESPGHAN) working group and the Cochrane Library database recommend oral rehydration therapy (ORT) and prompt realimentation for mild to moderate gastroenteritis (3,4).

**Vomiting in gastroenteritis and need for pharmacological treatment**

In the initial phase of viral AG, vomiting is a typical symptom (5). Thus, in AG caused by rotavirus infection during the first 1 to 3 days, repeated vomiting is present in 75% of children (6). Current practice recommendations for paediatric AG do not include pharmacologic treatment for vomiting (3). However, vomiting from gastroenteritis is distressing for patients and their families. In addition, vomiting is not only a direct cause of fluid loss but can also hamper successful oral rehydration therapy and it is a major factor of failure of ORT. Many physicians believe that vomiting is a contraindication to ORT. Physicians who provide care to paediatric patients in the emergency department consistently favour intravenous fluid therapy (IVT) for mild or moderate dehydration when vomiting is the major symptom (7,8). Thus, effective treatment of vomiting would lead to an important reduction in the use of IVT.

**Antiemetic agents and their use in clinical practice**

Various antiemetic agents have been used to prevent or reduce vomiting in children with gastroenteritis (9). The phenothiazines are dopamine antagonists and act centrally by blocking the chemoreceptor trigger zone. They are used to prevent or treat vomiting associated with drugs such as opiates, general anaesthetics, and cytotoxics. Unfortunately, severe dystonic reactions sometimes occur with phenothiazines, especially in children. Metoclopramide is a chlorinated procainamide derivative that acts primarily as a dopamine D2 receptor antagonist and has both central and peripheral actions. Metoclopramide also acts directly on the gastro-intestinal tract and it may be more effective than the phenothiazines for vomiting associated with gastro-duodenal disease. As with the phenothiazines, metoclopramide can induce acute dystonic reactions involving facial and skeletal muscle spasms and oculogyric crises. These dystonic effects are more common in paediatric age. In Italy metoclopramide use in children under 16 years old is not recommended because severe extrapyramidal reactions (10) were reported in case-control studies and case reports. Domperidone is a D2 receptor antagonist that acts on the chemoreceptor trigger zone. The medication also accelerates gastric emptying. Ondansetron is a specific 5HT3 antagonists which block 5HT3 receptors in the gastro-intestinal tract and in the central nervous system. It has been shown to be effective in the treatment of vomiting in patients receiving cytotoxic agents for cancer. Dexamethasone also has anti-emetic effects and is used to prevent vomiting associated with cancer chemotherapy. In this context it may be used alone or with other anti-emetics such as metoclopramide or a 5HT3 antagonist.

In the clinical practice antiemetic drugs are frequently used in children with gastroenteritis. A recent retrospective survey retrieved data from 4 national and international databases showed that prescription of antiemetic medication varied considerably (11). In particular, between 2% and 23% of children with gastroenteritis received prescriptions for antiemetic medications. The antihistamines dimenhydrinate and diphenhydramine were most frequently used in Germany and Canada, whereas promethazine was prescribed preferentially in the United States. In France, Spain, and Italy, the dopamine receptor antagonist domperidone was preferred as antiemetic treatment. Ondansetron was used in a minor proportion of antiemetic prescriptions. A recent survey carried out in Italy showed that 79% of participating clinicians prescribe antiemetic drugs to treat acute gastroenteritis (domperidone in primis followed by metoclopramide) (12). Data on Italian prescriptions collected by ARNO confirmed that among gastrointestinal agents, prokinetics (in 80% of cases domperidone) are the most prescribed in clinical practice (13).

**Antiemetic drugs in acute gastroenteritis: evidence of efficacy**

As demonstrated in 3 recently published meta-analysis, literature evaluating the efficacy of symptomatic drugs in reducing acute vomiting for AG in paediatric age is methodologically limited and focuses mainly on ondansetron (14-16). The 11 articles meeting the inclusion criteria evaluated various antiemetic agents (16): ondansetron (n=6), domperidone (n=2), metoclopramide (n=2), trimethobenzamide (n=2), pyrilamine-pentobarbital (n=2), dexamethasone (n=1), and promethazine (n=1). Six randomised controlled trials (RCTs) were carried out to evaluate ondansetron use in a total population of 745 children (17-22). All these studies compared ondansetron versus placebo. Furthermore two RCTs included the comparison of ondansetron to metoclopramide and dexamethasone (16). In three studies ondansetron was administrated per os and in the other three intravenous administration was preferred. Ondansetron, compared to placebo reduces the risk of future vomiting episodes (RR: 0.45; 95% CI: 0.33-0.62; Number Need to Treat, NNT=5), the number of patients needing intravenous rehydration (RR, 0.41; 95%CI: 0.28-0.62; NNT=5) and hospital admissions (RR, 0.52; 95% CI: 0.27-0.95; NNT=14) (16). The drug is not effective in reducing the access to ED for acute vomiting (14-16). With respect to side effects, except for the greater incidence of diarrhoea in patients receiving ondansetron treatment (16), no other significant differences between ondansetron and placebo were identified. A few studies were published regarding domperidone (23,24) and metoclopramide (21,23) and were characterized by small sample sizes, low methodological quality, and produced inconsistent results. These methodological issues do not allow to draw any conclusions about the risks/benefits balance of the two drugs (16). Furthermore, an adequate comparative evaluation between domperidone, metoclopramide and ondansetron is missing. All the studies included in the analysis were funded by pharmaceutical companies.

**The need for further evidence and potential transferability to clinical practice**

The above reported evidence shows the efficacy of the most recently registered molecule (ondansetron), but the studies were carried out on very heterogeneous populations for which risks, costs and benefits were not sufficiently assessed. The same evidence showed that a proper evaluation of anti-emetics drugs largely used in clinical practice (13), such as domperidone, is completely lacking. This lack of knowledge about antiemetics is particularly important considering the restricted indications for their use in Italy (this is particularly true for metoclopramide). In fact, these agents’ potential side effects as extra-pyramidal manifestations, lack of consciousness, convulsions are mostly dose-dependent and they have been addressed by a recent ad hoc report by AIFA paediatric commission (17).

In light of the above considerations, we propose a multicentre study comparing the efficacy of ondansetron and domperidone for the symptomatic treatment of vomiting in acute gastroenteritis. The study aims at answering the following clinical questions: a) would anti-emetics agents reduce the percentage of children who keep vomiting? b) would anti-emetics treatment favour the oral rehydration and reduce the need for nasogastric or intravenous fluid rehydration? c) would the treatment reduce the percentage of children accessing health services and needing hospital admission?

We believe that the results of such a trial could significantly impact current clinical practice. In fact it would define the real efficacy of domperidone largely used in clinical practice (despite the lack of a clear evidence-based assessment and a controversial safeness profile) compared to ondansetron whose use to treat vomiting in AG is not yet authorized in Italy despite evidence supporting its possible use. The design of this RCT, which closely reflect current clinical practice in EDs, should allow immediate transferability of results.

**OBJECTIVES**

- Primary Objective: To evaluate whether the oral administration of a symptomatic drug (ondansetron or domperidone) prevents intravenous or nasogastric rehydration in children vomiting and diagnosed acute gastroenteritis.
- Secondary Objective: To assess whether the oral administration of a symptomatic drug (ondansetron or domperidone) reduces the total duration and number of vomiting episodes and the need for hospital admission or ED access.

**METHODS**

**Study Design**

The study is a prospective, multicentre, double-blind randomized controlled trial. The study will be coordinated by the Institute for Maternal and Child Health - IRCCS Burlo Garofolo (Trieste) and by the Maternal and Child Health Laboratory of the Institute Mario Negri (Milan).

**Study Population**

The study will be conducted in hospital and the recruitment setting will be the paediatric emergency departments (ED). Children between 1 to 6 years will be included in the study.

Consecutive subjects accessing the ED during a 18 months period will be evaluated for inclusion/exclusion criteria by the doctor on call. The following inclusion and exclusion criteria will be used:

Inclusion Criteria

1) age from 1 to 6 years;

2) presumptive clinical diagnosis of acute gastroenteritis in patients with vomiting, with or without diarrhoea (see Annex 1 for AG clinical diagnosis and vomiting definitions);

3) more than three episodes of non-bilious, non-bloody vomiting within the previous 24 hours;

Exclusion Criteria:

1) treatment with antiemetics or antidiarrhoic drugs in the 6 hours prior to access to ED;

2) underlying chronic diseases (eg, malignancy, gastroesophageal reflux, migraine, renal failure, hypoalbuminemia, liver disease);

3) severe dehydration defined by a standard clinical score of >18 for children 12-24 months or >16 for children >24 months of age (see Annex 1) (20);

4) known hypersensitivity to ondansetron or domperidone;

5) previous enrolment in the study.

**Intervention**

After checking the inclusion and exclusion criteria, administration of oral rehydration solution (ORS) will be started following a standard protocol (see Annex 1 for definition). ORS will be prescribed by the doctor on call and administered under supervision of an emergency departments nurse.

In case of failure of the initial ORS administration (vomiting after ORS or fluid refusal), patients will be randomized to receive an oral administration of:

1) ondansetron syrup (0,15 mg/Kg of body weight);

2) domperidone syrup (0,5 mg/Kg of body weight);

3) placebo.

Children vomiting within 15 minutes after receiving the drug will be given a second dose.

After 45 to 60 minutes from treatment administration, a new attempt to administer ORS will be done, according to the standard protocol (see Annex 1).

After an adequate information of the study and before random allocation of subjects, written consent will be obtained by the doctor on call from the parents or legal guardian of children fulfilling the entry criteria and failing initial ORS administration. A register of all patients who were proposed to be enrolled in the study will be kept, and reason for refusal will be recorded.

A flow chart describing comparison groups and trial procedures is added in the Annex section (Annex 2).

**Outcomes**

Primary Outcome:

Percentage of patients needing nasogastric or intravenous rehydration after symptomatic oral treatment failure, defined as vomiting or fluid refusal after the second attempt of ORT.

Secondary Outcomes:

1) Percentage of subjects needing hospital admission for the same illness;

2) Percentage of subjects needing observation stay for more than 6 hours for the same illness;

3) Total emesis duration in the 3 allocation groups;

4) Number of episodes of vomiting in the 3 treatment groups during the follow-up period;

5) Percentage of subjects presenting adverse events.

Data regarding the study outcomes will be collected by doctors on call or bedside nurse during the patient stay in the ED (or any other paediatric unit devoted to short observation) using a standardized assessment tool. Patient will be reassessed at 30 minute intervals for a minimum of 6 hours. During the patients' hospital stay, doctors and nurses will be blind to treatment assignment. A telephone follow up call will be made 48 hours after ED discharge using a standard form by a research assistant blind to treatment assignment.

Note: We discussed whether to consider hospital admission as the primary study outcome. Unfortunately, criteria for hospital admission could differ in different hospitals and ED settings and various administrative typologies of hospital admission exist in the study centres, including the “short observation” which may vary across centres in the way it is defined and applied. A recent systematic review on ondansetron confirms that when considering hospitalization as an outcome, there is great heterogeneity. Authors indicate different criteria for hospital admission as a possible explanation (15). Definition and standardization of hospital admission criteria are difficult due to differences in the administrative and organizational characteristics among participating EDs. Furthermore, nasogastric or intravenous rehydration represent a more objective outcome and represent anyway a good proxy measure of hospital admission.

**Randomization**

Patients will be randomly assigned in fixed block size of nine to receive ondansetron or domperidone or placebo. Randomisation list will be generated using STATA software and will be stratified according to participating centres. Randomization procedure will be centralized and managed by an independent statistician at the Coordinating Centre. The randomization sequence will be provided to the central pharmacy, which will prepare and dispense to participating hospitals active drugs and placebo. After checking eligibility and failure of first ORS administration, the next available bag containing drug preparations will be opened by the doctor on call or by the bedside nurse and a weigh-appropriate dose will be administered to patients. All study investigators, personnel, and participants will be unaware of the randomization procedure and pharmaceutical preparations assignments.

**Blinding**

The pharmaceutical preparation will be directly sent to participating centres in closed, opaque and consecutive numbered bags by the central pharmacy. Drug preparations will be indistinguishable by taste, odour and appearance. All study investigators, personnel, and participants will be blind to preparations administered.

**Information retrieval**

A questionnaire detailing demographic data, medical history, allergies, history of present illness, and medication received will be collected at enrolment by doctors on call. Data relative to study outcomes will be collected by doctors on call or bedside nurse during the hospital stay. Patient will be reassessed at 30 minute intervals for a minimum of 6 hours and data will be collected at each assessment. Forty-eight hours after discharge, a blind researcher assistant will telephone the child's family to evaluate, using a standard form, the gastroenteritis evolution, the possible need of hospitalisation or readmission in ED and the final outcome.

Paper records will be transferred by each centre into electronic data base.

The percentage of subjects lost to follow up, based on available literature and taking into account the short follow up period, can be estimated as less than 10% (20,22).

**Sample size estimates**

Studies comparing ondansetron versus placebo are available in the literature while it was impossible to identify studies comparing ondansetron and domperidone. Two studies evaluating domperidone versus placebo were also identified, even if characterized by low methodological quality and small sample size. Based on the available literature, it appears plausible to hypothesize that ondansetron is more efficacious in comparison to placebo or domperidone and therefore design the study as a superiority trial. For sample size estimation we specifically referred to the Roslund RCT (22) that implemented a similar protocol to ours (enrolment of subjects with clinical acute gastroenteritis who failed initial ORS administration in the ED). Taking into account the above stringent eligibility criteria, we estimate that the enrolment of 540 children (i.e. 180 patients in each arm) will provide the study with a statistical power of 80% to detect a change from 50% in placebo group to 35% in domperidone group and 20% in ondansetron group in the proportion of children requiring nasogastric or intravenous rehydration, given a two-sided type I error of 0,05. Given the lack of available efficacy estimates, domperidone efficacy was estimated as intermediate between ondansetron and placebo.

**Statistical analysis**

Baseline characteristics of the three groups will be compared by the chi-square test for proportions and the analysis of variance or Kruskal-Wallis test (depending on data distribution) for continuous variables. Relative risks and 95% confidence intervals will be presented for categorical data while means and standard deviations for continuous data. For categorical outcomes, differences between groups will be evaluated using the chi-square test; for continuous outcomes using analysis of variance or Kruskal-Wallis test, depending on data distribution. Analyses will be performed with STATA software (version 9) according to the intention-to-treat principle. All p values will be two-sided, with a p value of less than 0.05 used to indicate statistical significance.

**Organizational Characteristics**

Coordinating Units: the study will be coordinated by the Department of Paediatrics, Institute for Maternal and Child Health - IRCCS Burlo Garofolo, Trieste (dr. Federico Marchetti) and by the Unit 1, Maternal and Child Health Laboratory, Institute Mario Negri, Milan (dr. Maurizio Bonati).

The study principal investigator will be dr. Federico Marchetti. The Coordinating Units will be responsible for the trial coordination (including training, randomization, study monitoring, data collection, data analysis, reporting).

Participating Units: subject will be enrolled in the following centres:

Unit 2: Pediatric Emergency Department, Azienda Ospedaliera - University of Padova (unit principal investigator: dr. Liviana Da Dalt)

Unit 3: Department of Pediatrics, Policlinico G.B. Rossi, University of Verona (unit principal investigator: dr. Enrico Valletta)

Unit 4: Emergency Department, Ospedale Pediatrico Bambino Gesú, IRCCS, Roma (unit principal investigator: dr. Antonino Reale)

Unit 5: Pediatric Emergency Department, P.O. Spedali Civili, Brescia (unit principal investigator: dr. Alberto Arrighini)

Unit 6: Department of Pediatrics, Azienda Policlinico, Modena (unit principal investigator: dr. Paolo Bertolani)

Unit 7: Pediatric Intensive Care Unit, Ospedale Civile Maggiore, Verona (unit principal investigator: dr. Paolo Biban)

Unit 8: Emergency Room and Emergency Medicine Division, G. Gaslini Institute, Genova (unit principal investigator: dr. Salvatore Renna, dr. Pasquale Di Pietro)

Unit 9: Department of Pediatrics, Azienda Ospedalieriera-University of Parma (unit principal investigator: dr. Maria Teresa Tondelli)

Unit 10: Emergency Department, Paediatric Hospital A. Meyer, Firenze (unit principal investigator: dr. Anna Pazzaglia, dr. Francesco Mannelli)

Unit 11: Emergency Department, Institute for Maternal and Child Health - IRCCS Burlo Garofolo, Trieste (unit principal investigator: dr. Gianni Messi)

Unit 12: Department of Pediatrics, Ospedale di Macerata (unit principal investigator: dr. Paolo Francesco Perri)

Unit 13: Emergency Department, Ospedale Infantile Regina Margherita di Torino (unit principal investigator: dr. Antonio Francesco Urbino)

Unit 14: Pediatric Emergency Department, Ospedale Giuseppe Moscati, Avellino (unit principal investigator: dr. Antonio Vitale)

Unit 15: Department of Pediatrics, Ospedale Castelli, Verbania (unit principal investigator: dr. Andrea Guala)

**Feasibility**

The Institute for Maternal and Child Health Burlo Garofolo is an IRCCS with a wide experience in carrying out clinical trails. In the Osservatorio Nazionale sulla Sperimentazione Clinica dei Medicinali (OsSC AIFA), 14 clinical trials carried out and coordinated by the Institute are recorded. Among those, two trials took place in EDs and one was funded by AIFA in the independent research grant 2006 (Randomized Controlled Double-Blind Vs. Placebo Multicentre Study On The Safety And Effectiveness Of Thalidomide In The Treatment Of Refractory Crohn’s Disease And Ulcerative Colitis In Children And Adolescents, Cod.: FARM64J2EB).

A Clinical Trial Quality Team (CTQT) has been established to guarantee a high quality of no profit clinical trials funded by the Institute. The CTQT establishment was notified to AIFA (Uffici Promozione e Ispezioni Good Clinical Practice) and the Institute has been included in the AIFA network for no profit clinical trial quality (“Rete delle strutture partecipanti al Progetto AIFA per la qualità nelle sperimentazioni no profit”).

A multidisciplinary (clinicians and epidemiologists) steering committee will establish to monitor the data, to ensure patient safety and to act as reference for Participants Units. Any problem arising during the trial will be discussed by committee members and decision will be shared with Participants Unit. The committee will be constituted by researchers who are either not directly involved in actual field work (dr. Federico Marchetti, dr. Maurizio Bonati) or are external to the study (dr. Luca Ronfani, epidemiologist, dr. Massimo Fontana, paediatric gastroenterologist).

**Timing**

A period of 24 months will be required to carry out the study. The recruitment period will last 18 months. Each patient will be recruited at the ED of the participating centres and followed up every 30 minutes at least for 6 hours. Each patient will be contacted by phone 48 hours after ED discharge to evaluate long-term outcomes. Every 6 months research meetings between Coordinating and Participating Units will be arranged to discuss research development and address any emerging issue. The final report will be prepared within the 24 month period.

**Good clinical practices**

The steering committee will monitor the safety and the overall quality and scientific integrity of the study. Every 6-8 months a monitor visits will be carried out in each centres by members of the steering committee.

**Ethical aspects**

The study will be approved by the ethics review boards of participating units of enrolment. All parents will be provided with appropriate information and a written informed consent form will be proposed to them.

**REFERENCES**

1. Malek MA, Curns AT, Holman RC, et al. Diarrhea- and rotavirus-associated hospitalizations among children less than 5 years of age: United States, 1997 and 2000. Pediatrics 2006;117(6):1887-1892.
2. Fontana M, Federico P, Cantucci S. Il ricovero pediatrico in Lombardia: cause, variabilità e possibile inappropriatezza. Medico e Bambino 2007;26:520-6
3. King CK, Glass R, Bresee JS, Duggan C. Managing acute gastroenteritis among children: oral rehydration, maintenance, and nutritional therapy. MMWR Recomm Rep 2003;52(RR-16):1-16.
4. Guarino A, Albano F, Ashkenazi S, et al., European Society for Paediatric Gastroenterology, Hepatology, and Nutrition/European Society for Paediatric Infectious Diseases evidence-based guidelines for the management of acute gastroenteritis in children in Europe: executive summary. J Pediatr Gastroenterol Nutr 2008;**46**:619–621
5. Elliott EJ. Acute gastroenteritis in children. BMJ 2007;334:35-40.
6. Bass ES, Pappano DA, Humiston SG. Rotavirus. Pediatr Rev 2007;28:183-91.
7. Ozuah PO, Avner JR, Stein RE. Oral rehydration, emergency physicians, and practice parameters: a national survey. Pediatrics 2002;109(2):259-261.
8. Reis EC, Goepp JG, Katz S, Santosham M. Barriers to use of oral rehydration therapy. Pediatrics 1994;93(5):708-711.
9. British National Formulary 54. London: BMJ Publishing Group Ltd; RPS Publishing; 2007.
10. Menniti-Ippolito F, Traversa G, Da Cas R, et al. Extrapyramidal reactions in children treated with metoclopramide. Ital J Pediatr 2004;30:49-52
11. Pfeil N, Uhlig U, Kostev K, et al. [Antiemetic medications in children with presumed infectious gastroenteritis--pharmacoepidemiology in Europe and Northern America.](http://www.ncbi.nlm.nih.gov/pubmed/18829050?ordinalpos=1&itool=EntrezSystem2.PEntrez.Pubmed.Pubmed_ResultsPanel.Pubmed_DefaultReportPanel.Pubmed_RVDocSum) J Pediatr 2008;153(5):659-62,
12. Albano F, Bruzzese E, Spagnuolo M, et al. Antiemetics for children with gastroenteritis: off label but still on in clinical practice. J Pediatr Gastroenterol Nutr 2006;43: 402-4.
13. AA.VV. Il profilo prescrittivo della popolazione pediatrica italiana nelle cure primarie. Gastrointestinali. Ricerca & Pratica 2004;120:267-72.
14. Alhashimi D, Alhashimi H, Fedorowicz Z. Antiemetics for reducing vomiting related to acute gastroenteritis in children and adolescents. Cochrane Database of Systematic Reviews 2006;4:CD005506
15. Szajewska H, Gieruszczak-Bialek D, Dylag M. Meta-analysis: ondansetron for vomiting in acute gastroenteritis in children. Aliment Pharmacol Ther 2007;25:393-400.
16. [DeCamp LR](http://www.ncbi.nlm.nih.gov/sites/entrez?Db=pubmed&Cmd=Search&Term="DeCamp LR"%5BAuthor%5D&itool=EntrezSystem2.PEntrez.Pubmed.Pubmed_ResultsPanel.Pubmed_DiscoveryPanel.Pubmed_RVAbstractPlus), [Byerley JS](http://www.ncbi.nlm.nih.gov/sites/entrez?Db=pubmed&Cmd=Search&Term="Byerley JS"%5BAuthor%5D&itool=EntrezSystem2.PEntrez.Pubmed.Pubmed_ResultsPanel.Pubmed_DiscoveryPanel.Pubmed_RVAbstractPlus), [Doshi N](http://www.ncbi.nlm.nih.gov/sites/entrez?Db=pubmed&Cmd=Search&Term="Doshi N"%5BAuthor%5D&itool=EntrezSystem2.PEntrez.Pubmed.Pubmed_ResultsPanel.Pubmed_DiscoveryPanel.Pubmed_RVAbstractPlus), [Steiner MJ](http://www.ncbi.nlm.nih.gov/sites/entrez?Db=pubmed&Cmd=Search&Term="Steiner MJ"%5BAuthor%5D&itool=EntrezSystem2.PEntrez.Pubmed.Pubmed_ResultsPanel.Pubmed_DiscoveryPanel.Pubmed_RVAbstractPlus). Use of antiemetic agents in acute gastroenteritis: a systematic review and meta-analysis. Arch Pediatr Adolesc Med 2008;162(9):858-865
17. Stork CM, Brown KM, Reilly TH, Secreti L, Brown LH. Emergency department treatment of viral gastritis using intravenous ondansetron or dexamethasone in children. Acad Emerg Med 2006;13(10):1027-33.
18. Reeves JJ, Shannon MW, Fleisher GR. Ondansetron decreases vomiting associated with acute gastroenteritis: a randomized, controlled trial. Pediatrics 2002; 109(4):e62.
19. Ramsook C, Sahagun-Carreon I, Kozinetz CA, Moro-Sutherland D. A randomized clinical trial comparing oral ondansetron with placebo in children with vomiting from acute gastroenteritis. Ann Emerg Med 2002;39(4):397-403.
20. Freedman SB, Adler M, Seshadri R, Powell EC. Oral ondansetron for gastroenteritis in a pediatric emergency department. N Engl J Med 2006;354(16):1698-1705.
21. Cubeddu LX, Trujillo LM, Talmaciu I, et al. Antiemetic activity of ondansetron in acute gastroenteritis. Aliment Pharmacol Ther 1997;11(1):185-191.
22. Roslund G, Hepps TS, McQuillen KK. The role of oral ondansetron in children with vomiting as a result of acute gastritis/gastroenteritis who have failed oral rehydration therapy: a randomized controlled trial. Ann Emerg Med 2008;52:22-29.
23. Van Eygen M, Dhondt F, Heck E, Ameryckx L, Van Ravensteyn H. A double-blind comparison of domperidone and metoclopramide suppositories in the treatment of nausea and vomiting in children. Postgrad Med J 1979;55(suppl 1) :36-39.
24. Dhondt F, Traen S, VanEygen M, Baran D, Willaert H. Domperidone (R-33812) suppositories: an effective antiemetic agent in diverse pediatric conditions: multi center trial. Curr Ther Res Clin Exp 1978;24:912-923.
25. Marchetti F, Santuccio C per il gruppo di lavoro sui Farmaci Pediatrico AIFA. Il Trattamento farmacologico del vomito: bilancio tra i rischi e benefici. Bif 2007;5:1-16 (inserto)

**Annex 1. Definitions**

**Clinical diagnosis of acute gastroenteritis**

We refer to the NICE guideline ([www.nice.org.uk/guidance/index.jsp?action=download&o=42316](http://www.nice.org.uk/guidance/index.jsp?action=download&o=42316)) that recommends:

"When considering a diagnosis of gastroenteritis, look for the following key characteristics:

- a recent change in stool consistency to loose or watery stools;
- recent onset of vomiting;
- recent contact with an individual with acute diarrhoea;
- exposure to known source of enteric infection (water or food borne);
- recent foreign travel.

Consider the following symptoms and signs as possible indicators of diagnoses other than gastroenteritis:

- high fever:

- age less than 3 months: > 38 °C

- age more than 3 months: > 39 °C.

- rapid breathing or laboured respirations
- altered conscious level (irritability, drowsiness)
- photophobia, neck stiffness and/or bulging fontanelle (in infants)
- non-blanching (haemorrhagic) rash
- blood and/or mucous in stool
- bilious vomiting (green)
- severe or localised abdominal pain
- abdominal distension or rebound tenderness."

**Vomiting**

According to NICE, we define vomiting as the forceful ejection of the stomach contents up to and out of the mouth ([www.nice.org.uk/guidance/index.jsp?action=download&o=42316](http://www.nice.org.uk/guidance/index.jsp?action=download&o=42316)).

Episodes separated by no more than two minutes are counted as a single episode. Non-productive retching, spilling of oral contents, and drooling were not considered vomiting (20).

**Study standard protocol of oral rehydration solution (ORS) administration[[2]](#footnote-3)**

**1^st^ hour:**

age 1 to 2 years:  50 cc of low osmolarity* ORS administered cold and in small, frequent volumes (small sips, time divided); this amount correspond to ½ coffee spoon (equivalent to 1,5 cc) every 2 minutes;

age 3 to 6 years:  100 cc of low osmolarity* ORS administered cold and in small, frequent volumes (small sips, time divided); this amount correspond to 1 coffee spoon (equivalent to 3-3,5 cc) every 2 minutes.

**From 2^nd^ to 6^th^ hour:**

Cold ORS administered at sips following the plan:

- mild dehydration: 30-60 ml/Kg body weight over 4-6 hours

- moderate dehydration: 60-90 ml/Kg body weight over 4-6 hours

* Low osmolarity ORS: sodium 60 mmol/L

**Dehydration clinical score**

|  | **Normal or mild dehydration**  **(1 Point)** | **Moderate dehydration**  **(2 Points)** | **Severe dehydration**  **(3 Points)** |
| --- | --- | --- | --- |
| Pinch-retraction time | Immediate | Slow (≤2 sec) | Very slow (>2 sec) |
| Feeling of skin to the touch | Normal | Dry | Clammy or cool |
| Condition of buccal mucosa | Moist | Dry | Very dry |
| Tears (if <24 months) | Present | Reduced | None |
| Heart rate | Within normal limits | Mild tachycardia (≤10% above normal) | Moderate tachycardia (>10% above normal) |
| Urine | Normal amount and color | Reduced amount or darker in color | None passed for >6 hr |
| Mental status | Thirsty, alert | Drowsy, irritable, restless | Limp, lethargic |

Severe dehydration defined by:

- children under 24 months of age: score >18

- children 24 months of age of older: score >16

**Annex 2. Study flow chart**

**Eligible subjects**

**(children attending ED)**

**Enrolled subjects**

**Inclusion/exclusion criteria**

Excluded if failure to meet inclusion criteria

Excluded if parental consent not given

**In case of vomiting or liquid refusal**

Excluded if ORT succeeds succeedssucceededss

**First oral rehydration solution (ORS) administration**

**Written parents consent**

**After 45-60 minutes from treatment administration 🡪 new ORT challenge**

**Oral Ondansetron**

0,15 mg/Kg of body weight

Centralised random allocation to a dose of

**Oral Domperidone**

0,5 mg/Kg of body weight

**Oral placebo**

**Outcomes evaluation**

**Outcomes evaluation**

**Outcomes evaluation**

Intention to treat analysis

Second dose given in case of vomiting within 15 minutes

Patients assessed at 30 minute intervals for a minimum of 6 hours of hospital stay. Telephone follow up at 48 hours after discharge

**Annex 3. Abbreviations**

AAP: American Academy of Pediatrics

AIFA: Agenzia Italiana del Farmaco (Italian Agency of Drugs)

AG: Acute gastroenteritis

ARNO: ARNO project (ON-LINE drug prescription monitoring system - global vision)

ED: emergency department

ESPGHAN: European Society for Paediatric Gastroenterology, Hepatology, and Nutrition

DRG: Diagnosis-related group

IRCCS: Istituto di Ricovero e Cura a Carattere Scientifico

IVT: intravenous fluid therapy

NICE: National Institute for Clinical Excellence

NNT: Number Need to Treat

ORS: oral rehydration solution

ORT: oral rehydration therapy

RCT: randomised controlled trial

WHO: World Health Organization

**2. Final study protocol**

**ORAL ONDANSETRON VS DOMPERIDONE FOR SYMPTOMATIC TREATMENT OF VOMITING DURING ACUTE GASTROENTERITIS IN CHILDREN: MULTICENTRE RANDOMIZED CONTROLLED TRIAL**

**No changes were made to the following sections:**

**BACKGROUND**

**OBJECTIVES**

**REFERENCES**

**Annexes**

Changes in Methods section are highlighted below:

**METHODS**

**Study Design**

The study is a prospective, multicentre, double-blind randomized controlled trial. The study will be coordinated by the Institute for Maternal and Child Health - IRCCS Burlo Garofolo (Trieste) and by the Maternal and Child Health Laboratory of the Institute Mario Negri (Milan).

**Study Population**

The study will be conducted in hospital and the recruitment setting will be the paediatric emergency departments (ED). Children between 1 to 6 years will be included in the study.

Consecutive subjects accessing the ED during a 18 months period will be evaluated for inclusion/exclusion criteria by the doctor on call. The following inclusion and exclusion criteria will be used:

Inclusion Criteria

1) age from 1 to 6 years;

2) presumptive clinical diagnosis of acute gastroenteritis in patients with vomiting, with or without diarrhoea (see Annex 1 for AG clinical diagnosis and vomiting definitions);

3) more than three episodes of non-bilious, non-bloody vomiting within the previous 24 hours;

Exclusion Criteria:

1) treatment with antiemetics or antidiarrhoic drugs in the 6 hours prior to access to ED;

2) underlying chronic diseases (eg, malignancy, gastroesophageal reflux, migraine, renal failure, hypoalbuminemia, liver disease);

3) severe dehydration defined by a standard clinical score of >18 for children 12-24 months or >16 for children >24 months of age (see Annex 1) (20);

4) known hypersensitivity to ondansetron or domperidone;

5) previous enrolment in the study

6) concomitant use of drugs that prolong the QT interval (i.e., macrolides, quinolones, ketoconazole, fluconazole, salmeterol, trimetoprim)

7) language barriers or inability to perform the telephone follow-up

**Intervention**

After checking the inclusion and exclusion criteria, administration of oral rehydration solution (ORS) will be started following a standard protocol (see Annex 1 for definition). ORS will be prescribed by the doctor on call and administered under supervision of an emergency departments nurse.

In case of failure of the initial ORS administration (vomiting after ORS or fluid refusal), patients will be randomized to receive an oral administration of:

1) ondansetron syrup (0,15 mg/Kg of body weight);

2) domperidone syrup (0,5 mg/Kg of body weight);

3) placebo.

Children vomiting within 15 minutes after receiving the drug will be given a second dose.

After 45 to 60 minutes from treatment administration, a new attempt to administer ORS will be done, according to the standard protocol (see Annex 1).

After an adequate information of the study and before random allocation of subjects, written consent will be obtained by the doctor on call from the parents or legal guardian of children fulfilling the entry criteria and failing initial ORS administration. A register of all patients who were proposed to be enrolled in the study will be kept, and reason for refusal will be recorded.

A flow chart describing comparison groups and trial procedures is added in the Annex section (Annex 2).

**Outcomes**

Primary Outcome:

Percentage of patients needing nasogastric or intravenous rehydration after symptomatic oral treatment failure, defined as vomiting or fluid refusal after the second attempt of ORT.

Secondary Outcomes:

1) Percentage of subjects needing hospital admission for the same illness;

2) Percentage of subjects needing observation stay for more than 6 hours for the same illness;

3) Total emesis duration in the 3 allocation groups;

4) Number of episodes of vomiting in the 3 treatment groups during the follow-up period;

5) Percentage of subjects presenting adverse events.

Data regarding the study outcomes will be collected by doctors on call or bedside nurse during the patient stay in the ED (or any other paediatric unit devoted to short observation) using a standardized assessment tool. Patient will be reassessed at 30 minute intervals for a minimum of 6 hours. During the patients' hospital stay, doctors and nurses will be blind to treatment assignment. A telephone follow up call will be made 48 hours after ED discharge using a standard form by a research assistant blind to treatment assignment.

Note: We discussed whether to consider hospital admission as the primary study outcome. Unfortunately, criteria for hospital admission could differ in different hospitals and ED settings and various administrative typologies of hospital admission exist in the study centres, including the “short observation” which may vary across centres in the way it is defined and applied. A recent systematic review on ondansetron confirms that when considering hospitalization as an outcome, there is great heterogeneity. Authors indicate different criteria for hospital admission as a possible explanation (15). Definition and standardization of hospital admission criteria are difficult due to differences in the administrative and organizational characteristics among participating EDs. Furthermore, nasogastric or intravenous rehydration represent a more objective outcome and represent anyway a good proxy measure of hospital admission.

**Randomization**

Patients will be randomly assigned in fixed block size of nine to receive ondansetron or domperidone or placebo. Randomisation list will be generated using STATA software and will be stratified according to participating centres. Randomization procedure will be centralized and managed by an independent statistician at the Coordinating Centre. The randomization sequence will be provided to the central pharmacy, which will prepare and dispense to participating hospitals active drugs and placebo. After checking eligibility and failure of first ORS administration, the next available bag containing drug preparations will be opened by the doctor on call or by the bedside nurse and a weigh-appropriate dose will be administered to patients. All study investigators, personnel, and participants will be unaware of the randomization procedure and pharmaceutical preparations assignments.

**Blinding**

The pharmaceutical preparation will be directly sent to participating centres in closed, opaque and consecutive numbered bags by the central pharmacy. Drug preparations will be indistinguishable by taste, odour and appearance. All study investigators, personnel, and participants will be blind to preparations administered.

**Information retrieval**

A questionnaire detailing demographic data, medical history, allergies, history of present illness, and medication received will be collected at enrolment by doctors on call. Data relative to study outcomes will be collected by doctors on call or bedside nurse during the hospital stay. Patient will be reassessed at 30 minute intervals for a minimum of 6 hours and data will be collected at each assessment. Forty-eight hours after discharge, a blind researcher assistant will telephone the child's family to evaluate, using a standard form, the gastroenteritis evolution, the possible need of hospitalisation or readmission in ED and the final outcome.

Paper records will be transferred by each centre into electronic data base.

The percentage of subjects lost to follow up, based on available literature and taking into account the short follow up period, can be estimated as less than 10% (20,22).

**Sample size estimates**

Studies comparing ondansetron versus placebo are available in the literature while it was impossible to identify studies comparing ondansetron and domperidone. Two studies evaluating domperidone versus placebo were also identified, even if characterized by low methodological quality and small sample size. Based on the available literature, it appears plausible to hypothesize that ondansetron is more efficacious in comparison to placebo or domperidone and therefore design the study as a superiority trial. For sample size estimation we specifically referred to the Roslund RCT (22) that implemented a similar protocol to ours (enrolment of subjects with clinical acute gastroenteritis who failed initial ORS administration in the ED). Taking into account the above stringent eligibility criteria, we estimate that the enrolment of 540 children (i.e. 180 patients in each arm) will provide the study with a statistical power of 80% to detect a change from 50% in placebo group to 35% in domperidone group and 20% in ondansetron group in the proportion of children requiring nasogastric or intravenous rehydration, given a two-sided type I error of 0,05. Given the lack of available efficacy estimates, domperidone efficacy was estimated as intermediate between ondansetron and placebo.

**Statistical analysis**

Baseline characteristics of the three groups will be compared by the chi-square test for proportions and the analysis of variance or Kruskal-Wallis test (depending on data distribution) for continuous variables. Relative risks and 95% confidence intervals will be presented for categorical data while means and standard deviations for continuous data. For categorical outcomes, differences between groups will be evaluated using the chi-square test; for continuous outcomes using analysis of variance or Kruskal-Wallis test, depending on data distribution. Analyses will be performed with STATA software (version 9) according to the intention-to-treat principle. All p values will be two-sided, with a p value of less than 0.05 used to indicate statistical significance.

Two interim analysis are planned, following the criteria defined by O'Brien-Fleming (Schulz KF. Lancet 2005;365:1657-61). The first interim analysis is planned at July 2013, two years after the enrolment of the first subject in the study. The interim stopping level (p value) for the difference between ondansetron vs domperidone/placebo is set at 0.0005. The second interim analysis is planned for November 4^th^ 2013 (the official closing date of the study) if: 1) the first analysis had not achieved the necessary significance, or 2) the initially estimated sample size had not been reached. The interim stopping level (p value) for the difference between ondansetron vs domperidone/placebo is set at 0.014.

**Organizational Characteristics**

Coordinating Units: the study will be coordinated by the Department of Paediatrics, Institute for Maternal and Child Health - IRCCS Burlo Garofolo, Trieste (dr. Federico Marchetti, dr. Luca Ronfani) and by the Unit 1, Maternal and Child Health Laboratory, Institute Mario Negri, Milan (dr. Maurizio Bonati).

The study principal investigator will be dr. Federico Marchetti. The Coordinating Units will be responsible for the trial coordination (including training, randomization, study monitoring, data collection, data analysis, reporting).

Dr. Francesca Rovere, dr. Alessandra Maestro and dr. Davide Zanon will be responsible for monitoring drug production and distribution.

Study Secretary will be coordinated by dr. Alessia Fratte (tel: 040 3785401, fax: 040 3785260, e-mail: [sondo@burlo.trieste.it](mailto:sondo@burlo.trieste.it)).

Participating Units: subject will be enrolled in the following centres:

Unit 2: Pediatric Emergency Department, Azienda Ospedaliera - University of Padova (unit principal investigator: dr. Tiziana Zangardi)

Unit 3: Department of Pediatrics, Policlinico G.B. Rossi, University of Verona (unit principal investigator: dr. Enrico Valletta)

Unit 4: Emergency Department, Ospedale Pediatrico Bambino Gesú, IRCCS, Roma (unit principal investigator: dr. Antonino Reale)

Unit 5: Pediatric Emergency Department, P.O. Spedali Civili, Brescia (unit principal investigator: dr. Alberto Arrighini)

Unit 6: Department of Pediatrics, Azienda Policlinico, Modena (unit principal investigator: dr. Paolo Bertolani)

Unit 7: Pediatric Intensive Care Unit, Ospedale Civile Maggiore, Verona (unit principal investigator: dr. Paolo Biban)

Unit 8: Emergency Room and Emergency Medicine Division, G. Gaslini Institute, Genova (unit principal investigator: dr. Salvatore Renna, dr. Pasquale Di Pietro)

Unit 9: Department of Pediatrics, Azienda Ospedalieriera-University of Parma (unit principal investigator: dr. Maria Teresa Tondelli)

Unit 10: Emergency Department, Paediatric Hospital A. Meyer, Firenze (unit principal investigator: dr. Anna Pazzaglia, dr. Francesco Mannelli)

Unit 11: Emergency Department, Institute for Maternal and Child Health - IRCCS Burlo Garofolo, Trieste (unit principal investigator: dr Egidio Barbi, dr. Gianni Messi)

Unit 12: Department of Pediatrics, Ospedale di Macerata (unit principal investigator: dr. Paolo Francesco Perri)

Unit 13: Emergency Department, Ospedale Infantile Regina Margherita di Torino (unit principal investigator: dr. Antonio Francesco Urbino)

Unit 14: Pediatric Emergency Department, Ospedale Giuseppe Moscati, Avellino (unit principal investigator: dr. Antonio Vitale)

Unit 15: Department of Pediatrics, Ospedale Castelli, Verbania (unit principal investigator: dr. Andrea Guala)

Unit 16: Department of Pediatrics, Treviso Hospital (unit principal investigator: dr. Liviana Da Dalt)

Unit 17: Department of Pediatrics, Ospedale Maggiore, Bologna (unit principal investigator: dr. Elisa Mazzoni)

**Drugs production and supply**

Drugs manufacture will be committed by the Coordinating Centre to Monteresearch S.r.l. (via IV Novembre 92, Bollate, Milano), a pharmaceutical laboratory licensed by the Italian Medicines Agency (AIFA) to produce and manage medical products for clinical trials according to Good Manufacture Practice. All the manufacturing operations will be developed and agreed with the Clinical Trial Service and Clinical Trial Quality Team of the Coordinating Centre. Monteresearch will dispense active drugs and placebo directly to each participating centre.

**Feasibility**

The Institute for Maternal and Child Health Burlo Garofolo is an IRCCS with a wide experience in carrying out clinical trails. In the Osservatorio Nazionale sulla Sperimentazione Clinica dei Medicinali (OsSC AIFA), 14 clinical trials carried out and coordinated by the Institute are recorded. Among those, two trials took place in EDs and one was funded by AIFA in the independent research grant 2006 (Randomized Controlled Double-Blind Vs. Placebo Multicentre Study On The Safety And Effectiveness Of Thalidomide In The Treatment Of Refractory Crohn’s Disease And Ulcerative Colitis In Children And Adolescents, Cod.: FARM64J2EB).

A Clinical Trial Quality Team (CTQT) has been established to guarantee a high quality of no profit clinical trials funded by the Institute. The CTQT establishment was notified to AIFA (Uffici Promozione e Ispezioni Good Clinical Practice) and the Institute has been included in the AIFA network for no profit clinical trial quality (“Rete delle strutture partecipanti al Progetto AIFA per la qualità nelle sperimentazioni no profit”).

A multidisciplinary (clinicians and epidemiologists) steering committee will establish to monitor the data, to ensure patient safety and to act as reference for Participants Units. Any problem arising during the trial will be discussed by committee members and decision will be shared with Participants Unit. The committee will be constituted by researchers who are either not directly involved in actual field work (dr. Federico Marchetti, dr. Maurizio Bonati) or are external to the study (dr. Luca Ronfani, epidemiologist, dr. Massimo Fontana, paediatric gastroenterologist).

**Timing**

A period of 24 months will be required to carry out the study. The recruitment period will last 18 months. Each patient will be recruited at the ED of the participating centres and followed up every 30 minutes at least for 6 hours. Each patient will be contacted by phone 48 hours after ED discharge to evaluate long-term outcomes. Every 6 months research meetings between Coordinating and Participating Units will be arranged to discuss research development and address any emerging issue. The final report will be prepared within the 24 month period.

**Good clinical practices**

The steering committee will monitor the safety and the overall quality and scientific integrity of the study. Every 6-8 months a monitor visits will be carried out in each centres by members of the steering committee.

**Opening of the blind**

The opening of the blind will be allowed in the presence of one of the following criteria:

1. appearance of a serious, unexpected adverse event;

2. need to administered a antiemetic drug for significant worsening of vomiting.

**Ethical aspects**

The study will be approved by the ethics review boards of participating units of enrolment. All parents will be provided with appropriate information and a written informed consent form will be proposed to them.

**3. Summary of changes**

| **Changes made to the original protocol** | **Date of approval of the changes by the Ethic Committee of the Coordinating Centre*** |
| --- | --- |
| **Methods section, Study population, Exclusion criteria**.  Two exclusion criteria were added:  "6) concomitant use of drugs that prolong the QT interval (i.e., macrolides, quinolones, ketoconazole, fluconazole, salmeterol, trimetoprim)  7) language barriers or inability to perform the telephone follow-up." | 02.05.2011 |
| **Methods section, Statistical analyses**  Two interim analysis were planned:  " Two interim analysis are planned, following the criteria defined by O'Brien-Fleming (Schulz KF. Lancet 2005;365:1657-61). The first interim analysis is planned at July 2013, two years after the enrolment of the first subject in the study. The interim stopping level (p value) for the difference between ondansetron vs domperidone/placebo is set at 0.0005. The second interim analysis is planned for November 4^th^ 2013 (the official closing date of the study) if: 1) the first analysis had not achieved the necessary significance, or 2) the initially estimated sample size had not been reached. The interim stopping level (p value) for the difference between ondansetron vs domperidone/placebo is set at 0.014." | 20.06.2013 |
| **Methods section, Organizational Characteristics**  More details on personnel involved in the study at the Coordinating Centre were added; the principal investigators of Unit 2 and Unit 11 were changed; two more participating Units were added:  "Unit 16: Department of Pediatrics, Treviso Hospital (unit principal investigator: dr. Liviana Da Dalt)  Unit 17: Department of Pediatrics, Ospedale Maggiore, Bologna (unit principal investigator: dr. Elisa Mazzoni)" | 02.05.2011 |
| **Methods section.**  A paragraph on drugs production was added:  "**Drugs production and supply**  Drugs manufacture will be committed by the Coordinating Centre to Monteresearch S.r.l. (via IV Novembre 92, Bollate, Milano), a pharmaceutical laboratory licensed by the Italian Medicines Agency (AIFA) to produce and manage medical products for clinical trials according to Good Manufacture Practice. All the manufacturing operations will be developed and agreed with the Clinical Trial Service and Clinical Trial Quality Team of the Coordinating Centre. Monteresearch will dispense active drugs and placebo directly to each participating centre." | 02.05.2011 |
| **Methods section.**  A paragraph on the opening of the blind was added:  **"Opening of the blind**  The opening of the blind will be allowed in the presence of one of the following criteria:  1. appearance of a serious, unexpected adverse event;  2. need to administered a antiemetic drug for significant worsening of vomiting." | 02.05.2011 |

* All changes were submitted and approved by the Ethic Committee of all the participating centers.

1. Published on BMC Pediatrics:

   Marchetti F et al. Oral ondansetron versus domperidone for symptomatic treatment of vomiting during acute gastroenteritis in children: multicentre randomized controlled trial. BMC Pediatr 2011;11:15. [↑](#footnote-ref-2)
2. This standard protocol is the result of the combination of international guidelines recommendations and study committee consensus derived from ED clinical practice. [↑](#footnote-ref-3)
