# Supplementary material for: Oral Ondansetron versus Domperidone for Acute Gastroenteritis in Pediatric Emergency Departments: Multicenter Double Blind Randomized Controlled Trial
Source: PLoS One. 2016 Nov 23;11(11):e0165441. doi: 10.1371/journal.pone.0165441 (PMC5120790; doi:10.1371/journal.pone.0165441)
Supplement: S1 Table — (DOC) [file pone.0165441.s004.doc]

**S1 Table**

**Dehydration clinical score**

|  | **Normal or mild dehydration**  **(1 Point)** | **Moderate dehydration**  **(2 Points)** | **Severe dehydration**  **(3 Points)** |
| --- | --- | --- | --- |
| Pinch-retraction time | Immediate | Slow (≤2 sec) | Very slow (>2 sec) |
| Feeling of skin to the touch | Normal | Dry | Clammy or cool |
| Condition of buccal mucosa | Moist | Dry | Very dry |
| Tears (if <24 months) | Present | Reduced | None |
| Heart rate | Within normal limits | Mild tachycardia (≤10% above normal) | Moderate tachycardia (>10% above normal) |
| Urine | Normal amount and color | Reduced amount or darker in color | None passed for >6 hr |
| Mental status | Thirsty, alert | Drowsy, irritable, restless | Limp, lethargic |

Severe dehydration defined by:

- children under 24 months of age: score >18

- children 24 months of age of older: score >16
