# Supplementary material for: Oral Ondansetron versus Domperidone for Acute Gastroenteritis in Pediatric Emergency Departments: Multicenter Double Blind Randomized Controlled Trial
Source: PLoS One. 2016 Nov 23;11(11):e0165441. doi: 10.1371/journal.pone.0165441 (PMC5120790; doi:10.1371/journal.pone.0165441)
Supplement: S2 Table — (DOC) [file pone.0165441.s005.doc]

**S2 Table**

**Interim analyses results for the main study outcome (subjects needing nasogastric or intravenous rehydration)**

**S2 Table A. First interim analysis (01 July 2013). O'Brien-Fleming criteria to stop the trial: p=0.0005**

| **Ondansetron (n=110)**  **n (%)** | **Domperidone**  **(n=109)**  **n (%)** | **Placebo**  **(n=109)**  **n (%)** | **Ondansetron vs Domperidone**  **RR (99.95% CI)**  **p*** | **Ondansetron vs**  **Placebo**  **RR (99.95% CI)**  **p*** |
| --- | --- | --- | --- | --- |
| 11 (10.0%) | 28 (25.7%) | 31 (28.4%) | 0.39 (0.12 to 1.22)  p=0.002 | 0.35 (0.11 to 1.09)  p=0.0005 |

**S2 Table B. Second interim analysis (04 November 2013). O'Brien-Fleming criteria to stop the trial: p=0.014**

|  | | | | |
| --- | --- | --- | --- | --- |
| **Ondansetron (n=119)**  **n (%)** | **Domperidone**  **(n=119)**  **n (%)** | **Placebo**  **(n=118)**  **n (%)** | **Ondansetron vs Domperidone**  **RR (98.6% CI)**  **p*** | **Ondansetron vs**  **Placebo**  **RR (98.6% CI)**  **p*** |
| 14 (11.8%) | 30 (25.2%) | 34 (28.8%) | 0.47 (0.23 to 0.97) p=0.008 | 0·41 (0.20 to 0.83) p=0.001 |

* Chi square test
