# Supplementary material for: Oral Ondansetron versus Domperidone for Acute Gastroenteritis in Pediatric Emergency Departments: Multicenter Double Blind Randomized Controlled Trial
Source: PLoS One. 2016 Nov 23;11(11):e0165441. doi: 10.1371/journal.pone.0165441 (PMC5120790; doi:10.1371/journal.pone.0165441)
Supplement: S3 Table — (DOC) [file pone.0165441.s006.doc]

**S3 Table**

**Adverse events**

|  | **Domperidone**  **(n=119)** | **Ondansetron**  **(n=119)** | **Placebo**  **(n=118)** | **p** |
| --- | --- | --- | --- | --- |
| Adverse events during ED stay, n (%) | 1 (0.8%) | 2 (1.7%) | 0 | 0.4 |
| Adverse events in the 48 hours follow up period, n (%) | 4 (3.4%) | 4 (3.3%) | 2 (1.7%) | 0.7 |
| Total adverse events, n (%) | 5 (4.2%) | 6 (5.0%) | 2 (1.7%) | 0.4 |
| - increase in drowsiness, asthenia or irritability | 2 | 2 | 1 |  |
| - diarrhea/abdominal pain | 2 | 3 | 1 |  |
| - headache | 1 | 1 | - |  |
