# Supplementary material for: Oral Ondansetron versus Domperidone for Acute Gastroenteritis in Pediatric Emergency Departments: Multicenter Double Blind Randomized Controlled Trial
Source: PLoS One. 2016 Nov 23;11(11):e0165441. doi: 10.1371/journal.pone.0165441 (PMC5120790; doi:10.1371/journal.pone.0165441)
Supplement: S1 Text — (DOC) [file pone.0165441.s007.doc]

**S1 Text**

**Definitions of acute gastroenteritis and vomiting**

**Clinical diagnosis of acute gastroenteritis**

We refer to the NICE guideline ([www.nice.org.uk/guidance/index.jsp?action=download&o=42316](http://www.nice.org.uk/guidance/index.jsp?action=download&o=42316)) that recommends:

"When considering a diagnosis of gastroenteritis, look for the following key characteristics:

a recent change in stool consistency to loose or watery stools;

- recent onset of vomiting;

- recent contact with an individual with acute diarrhoea;

- exposure to known source of enteric infection (water or food borne);

- recent foreign travel.

Consider the following symptoms and signs as possible indicators of diagnoses other than gastroenteritis:

- high fever:

- age less than 3 months: > 38 °C

- age more than 3 months: > 39 °C.

- rapid breathing or laboured respirations

- altered conscious level (irritability, drowsiness)

- photophobia, neck stiffness and/or bulging fontanelle (in infants)

- non-blanching (haemorrhagic) rash

- blood and/or mucous in stool

- bilious vomiting (green)

- severe or localised abdominal pain

- abdominal distension or rebound tenderness."

**Definition of vomiting**

According to NICE, we define vomiting as the forceful ejection of the stomach contents up to and out of the mouth ([www.nice.org.uk/guidance/index.jsp?action=download&o=42316](http://www.nice.org.uk/guidance/index.jsp?action=download&o=42316)).

Episodes separated by no more than two minutes are counted as a single episode. Non-productive retching, spilling of oral contents, and drooling were not considered vomiting.
