# Supplementary material for: Oral Ondansetron versus Domperidone for Acute Gastroenteritis in Pediatric Emergency Departments: Multicenter Double Blind Randomized Controlled Trial
Source: PLoS One. 2016 Nov 23;11(11):e0165441. doi: 10.1371/journal.pone.0165441 (PMC5120790; doi:10.1371/journal.pone.0165441)
Supplement: S2 Text — (DOC) [file pone.0165441.s008.doc]

**S2 Text**

**Study standard protocol of oral rehydration solution (ORS) administration**

This standard protocol is the result of the combination of international guidelines recommendations and study committee consensus derived from ED clinical practice:

**1st hour**:

age 1 to 2 years:  50 cc of low osmolarity* ORS administered cold and in small, frequent volumes (small sips, time divided); this amount correspond to ½ coffee spoon (equivalent to 1.5 cc) every 2 minutes;

age 3 to 6 years:  100 cc of low osmolarity* ORS administered cold and in small, frequent volumes (small sips, time divided); this amount correspond to 1 coffee spoon (equivalent to 3-3.5 cc) every 2 minutes.

**From 2nd to 6th hour**:

Cold ORS administered at sips following the plan:

- mild dehydration: 30-60 ml/Kg body weight over 4-6 hours

- moderate dehydration: 60-90 ml/Kg body weight over 4-6 hours

* Low osmolarity ORS: sodium 60 mmol/L
